# Supplementary material for: Aircraft surveys for air eDNA: probing biodiversity in the sky
Source: PeerJ. 2023 Apr 14;11:e15171. doi: 10.7717/peerj.15171 (PMC10108859; doi:10.7717/peerj.15171)
Supplement: Supplemental Information 3 — (A) Genetic aerial survey grid perimeter denoting [1] rural-agricultural land in purple overlay and [2] urban-suburban populated area in red overlay under superimposed flight track (blue) from Research Flight #1 at 300 m; (B) close-up view of putative [1] rural-agricultural aerosolization sources representative of the overall surveyed area; and putative (C) close-up view of putative [2] populated urban-suburban aerosolization sources in the surveyed area. Note: This representation approximates bioaerosol (environmental DNA) sources, and facilities could be active or inactive at time of flight. Emissions sources not represented could include fertilizer application, landfills, smoke, and construction. Map credit: © Google Landsat/ Copernicus. [file peerj-11-15171-s003.pdf]

Supplemental Information

File S1: Ground level major sources of bioaerosols from rural-agricultural emissions/farming operations and urban-suburban activities in the study area: (**A**) Genetic aerial survey grid perimeter denoting [1] rural-agricultural land in purple overlay and [2] urban-suburban populated area in red overlay under superimposed flight track (blue) from Research Flight #1 at 300 m; (**B**) close-up view of putative [1] rural-agricultural aerosolization sources representative of the overall surveyed area; and putative (**C**) close-up view of putative [2] populated urban-suburban aerosolization sources in the surveyed area. Note: This representation approximates bioaerosol (environmental DNA) sources, and facilities could be active or inactive at time of flight. Emissions sources not represented could include fertilizer application, landfills, smoke, and construction. Map credit: © Google Landsat/ Copernicus.

A

Legend

Waste treatment
Hospital-medical

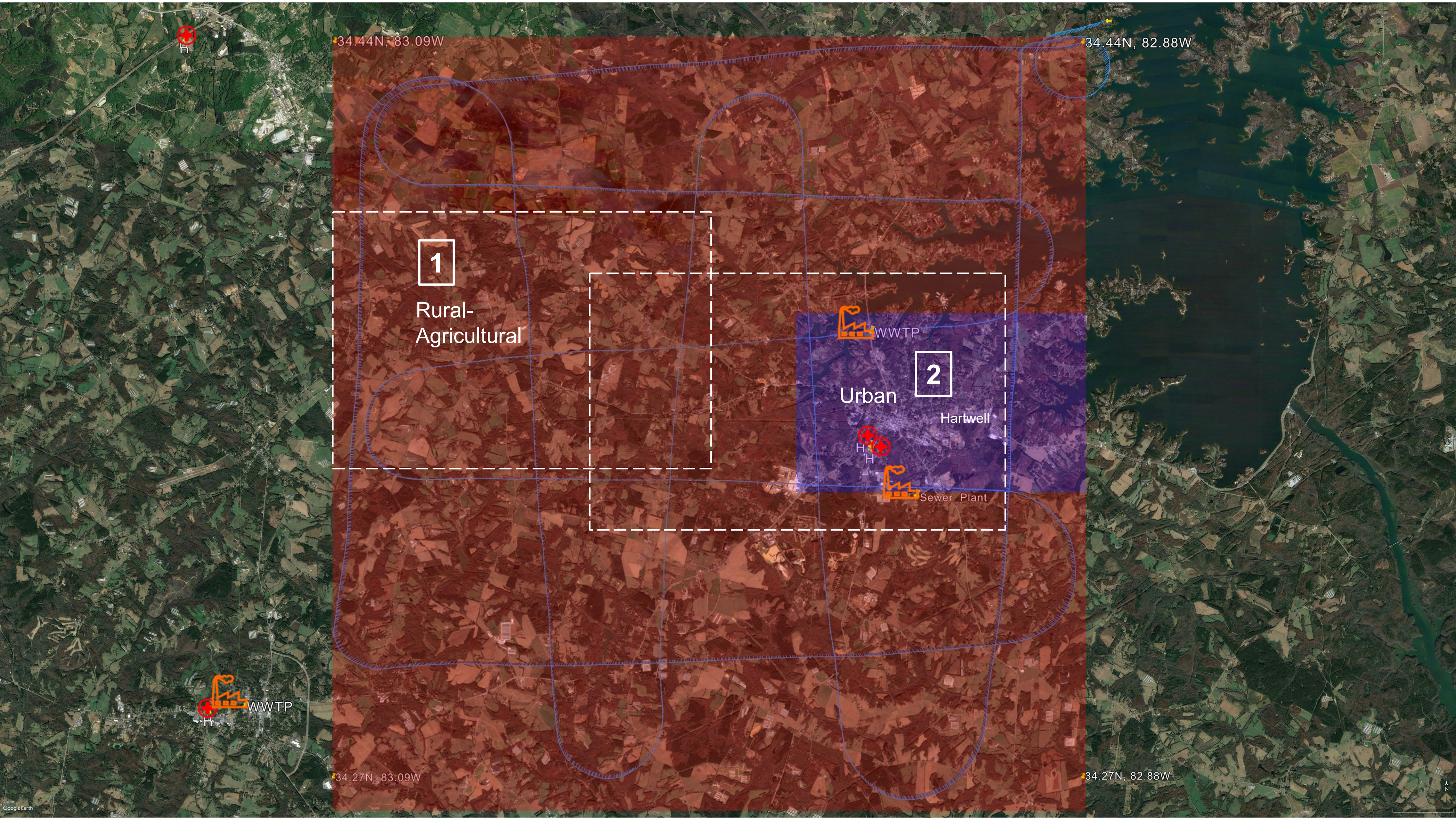

B

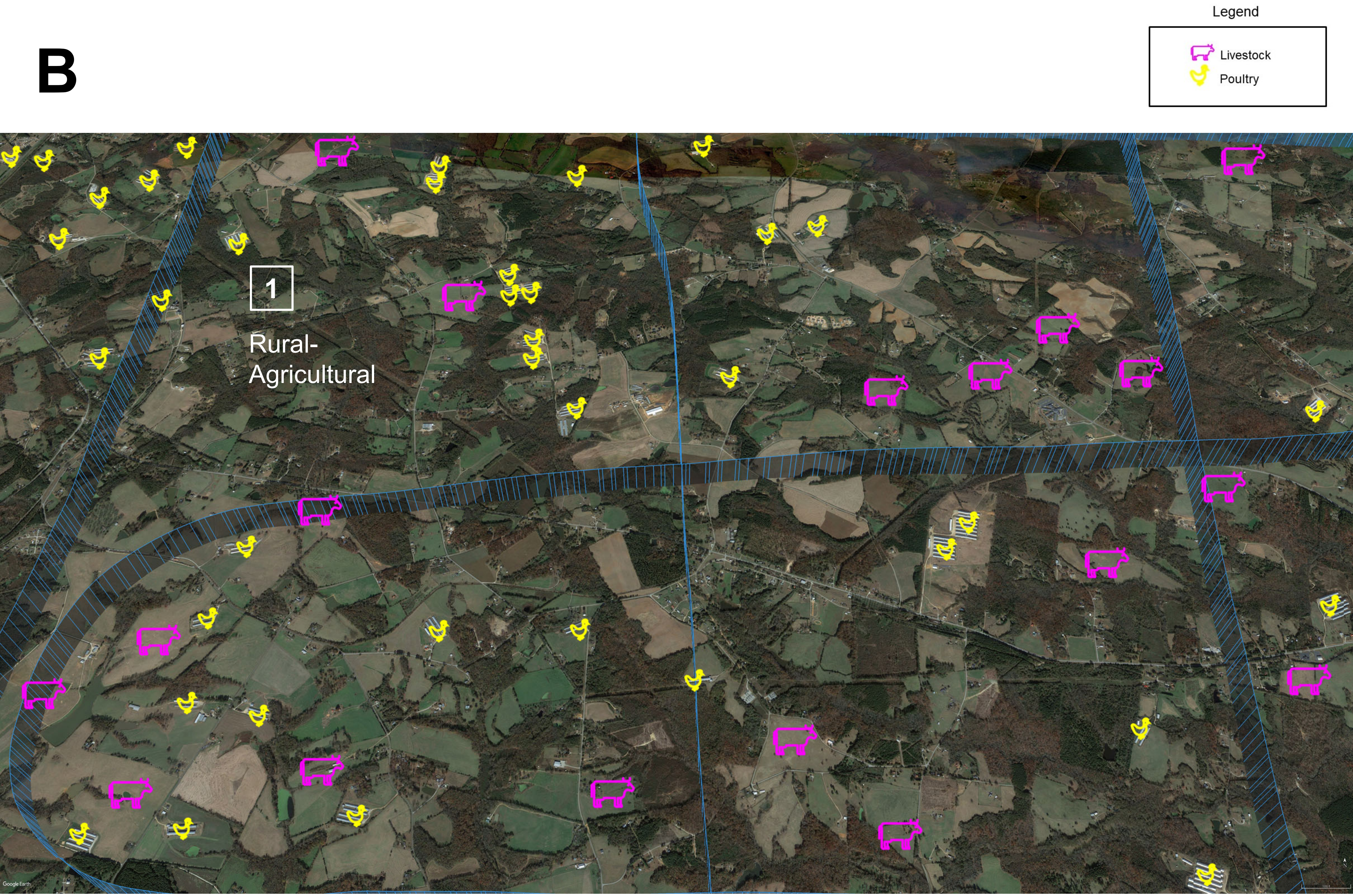
Legend
Livestock
Poultry
1
Rural-
Agricultural
Google Earth

C

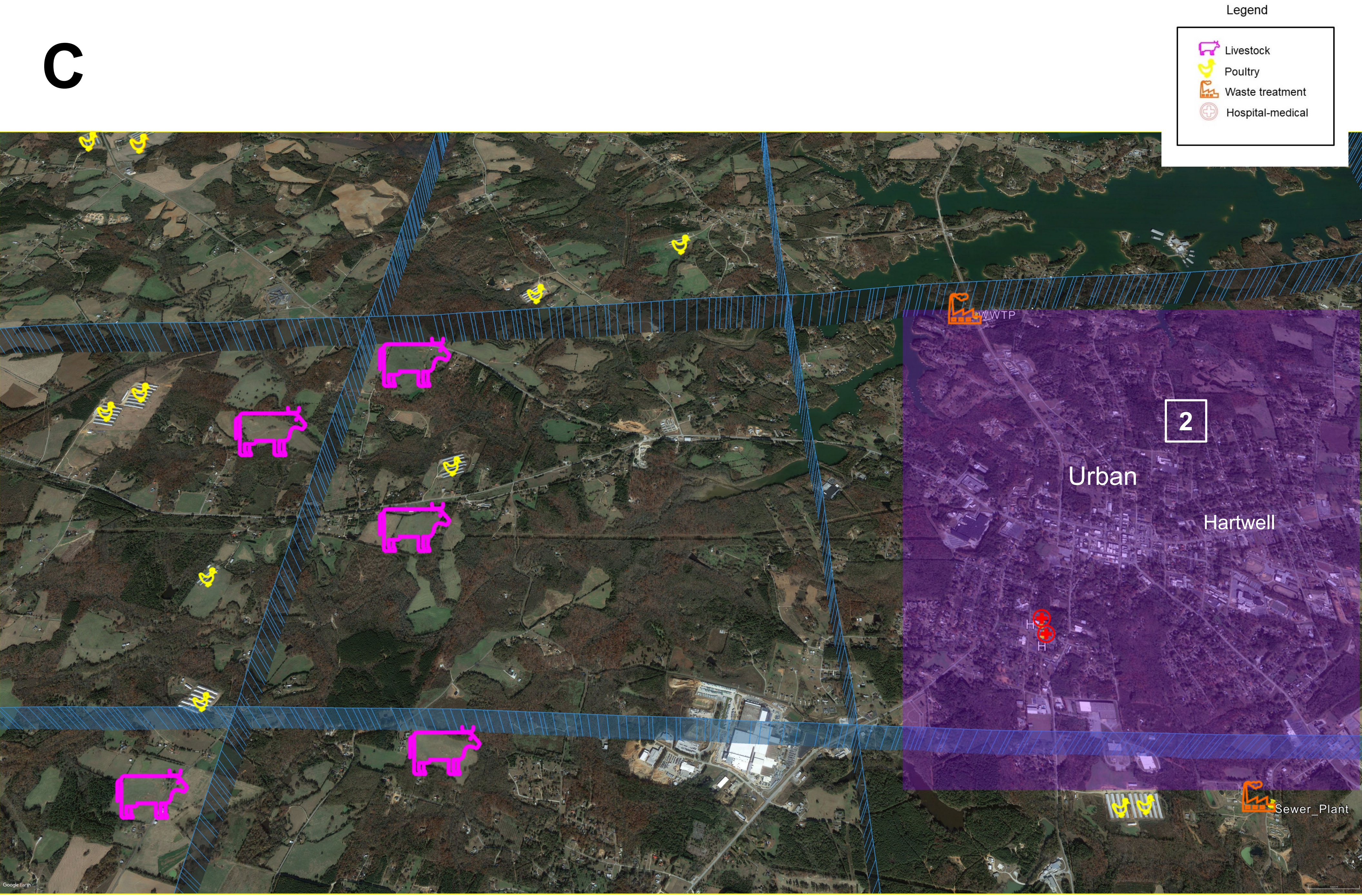

Legend
Livestock
Poultry
Waste treatment
Hospital-medical
WWTP
2
Urban
Hartwell
H
H
Sewer_Plant
Google Earth
