# Supplementary material for: Aircraft surveys for air eDNA: probing biodiversity in the sky
Source: PeerJ. 2023 Apr 14;11:e15171. doi: 10.7717/peerj.15171 (PMC10108859; doi:10.7717/peerj.15171)
Supplement: Supplemental Information 4 [file peerj-11-15171-s004.pdf]

Supplemental Information

Data S1.1

Preliminary Test Flights: Sampler performance data

Test Flight #	Date	Altitude ft MSL (m AGL)	Ave Flow, D1 (L·min^{-1})	Ave Flow, D2 (L·min^{-1})	Pressure, D1 (-kPa)	Pressure, D2 (-kPa)	Total Air Volume Filtered† L (m^3)
Test_1	4-Apr	1800 (300)	16		54		1,472 (1.472)
Test_2	2-May	1800 (300)	15	14	52	48	2,175 (2.175)

Data S1.1 Table 1. Sampler performance data for pre-study instrumentation test flights prior to surveys. MSL: mean sea level, AGL: above ground level, Ave: average, D1/D2: duplicate 1/duplicate 2. †Total air volume was calculated as the product of airflow × sampling time. Note: No duplicate sample was taken on the 4-Apr test flight as we had built only one probe at the time.

Preliminary Test Flights: Meteorological data

**Test Flight #**	**Date**	**Sampling time local** (duration in hhmm)	**METAR_Zulu** (UTC)	**Wind Direction** (°)	**Wind Velocity** (kts)	**Visibility** (SM)	**Sky Cdn**	**Air Temp** (°C)	**Dewpoint** (°C)	**Altimeter** (in-Hg)	**Density Altitude** (ft)	**Temp at Alt** (°C)
Test_1	4-Apr	1851-2053 (0132)	2232	290	04	10	clear	21	02	2995	1700	11
Test_2	2-May	1901-2016 (0115)	2254	240	08	10	clear	29	16	3000	2500	13

**Test Flight #**	**Date**	**Altitude** ft MSL (m AGL)	**Ave GS** (kts)	**Distance** (NM)	**Remarks**
Test_1	4-Apr	1800 (300)	89	134	Wind WNW (at alt)
Test_2	2-May	1800 (300)	92	107	Smoke indicated wind SW

Data S1.1 Table 2. Aeronautical and meteorological data for preliminary test flights: METAR: meteorological aerodrome; SM: statute miles, MSL: mean sea level, AGL: above ground level, GS: aircraft ground speed, NM: nautical miles, WTA: winds and temperatures aloft. Note: The wind direction given indicates that the wind comes in front of the aircraft when its heading equals the wind direction.

Surface Analysis Prognostic Chart Forecasts (Continental US) for Instrumentation Test Flights	
4-Apr	2-May
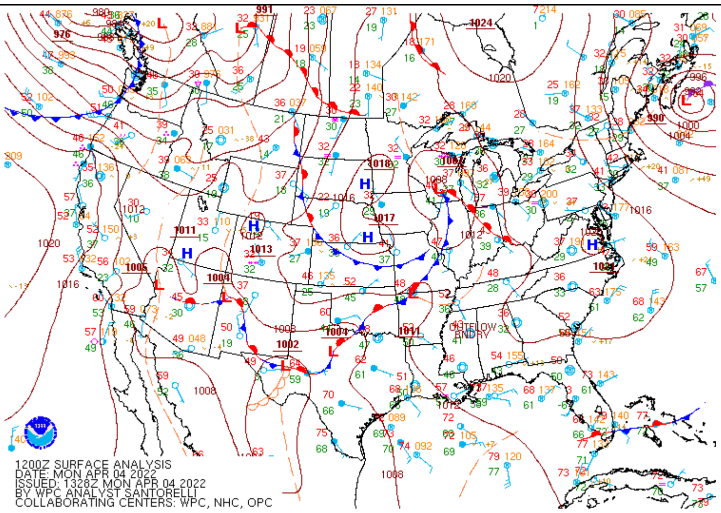	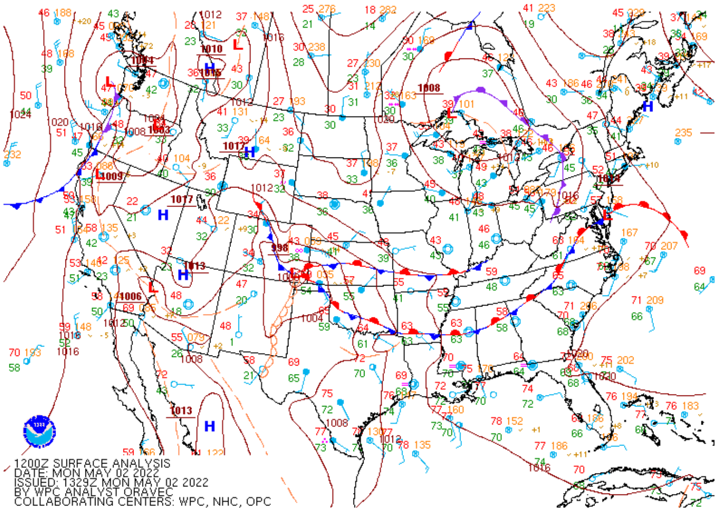

Data S1.1 Figure 1. Surface analysis prognostic chart forecasts for preliminary test flights conducted prior to research surveys: Surface analysis credit: © National Oceanic and Atmospheric Administration (NOAA).

Test_1 (4-Apr)	AND,2022-04-04 22:30,M,M,M,290.00,4.00,M,29.95,M,10.00,M,CLR,KAND 042230Z AUTO 29004KT 10SM CLR 21/02 A2995 RMK T02100020<br>18A,2022-04-04 23:15,69.10,33.40,26.64,230.00,4.00,0.00,29.95,M,10.00,M,CLR,K18A 042315Z AUTO 23004KT 10SM CLR 21/01 A2995 RMK AO2 T02060008
Test_2 (2-May)	AND,2022-05-02 22:55,M,M,M,230.00,8.00,M,30.00,M,10.00,M,CLR,KAND 022255Z AUTO 23008KT 10SM CLR 29/16 A3000 RMK T02900160<br>18A,2022-05-02 23:35,79.70,60.80,52.48,0.00,0.00,0.00,30.02,M,10.00,M,CLR,K18A 022335Z AUTO 00000KT 10SM CLR 26/16 A3002 RMK AO2 T02650160

Data S1.1 Table 3. Meteorological aerodrome (METAR) data from automated surface [weather] observation systems (ASOS) for both preliminary test flights originating out of AND, Anderson Regional Airport (Anderson, SC). *Data are provided in the following order: station, valid, tmpf, dwpf, relh, drct, sknt, p01i, alti, mslp, vsby, gust, skyc, metar; m=missing.* Airport meteorological automated surface weather observations (ASOS) METAR data codes can be referenced from NOAA at https://www.weather.gov/media/asos/aum-toc.pdf (accessed 4 August 2022).

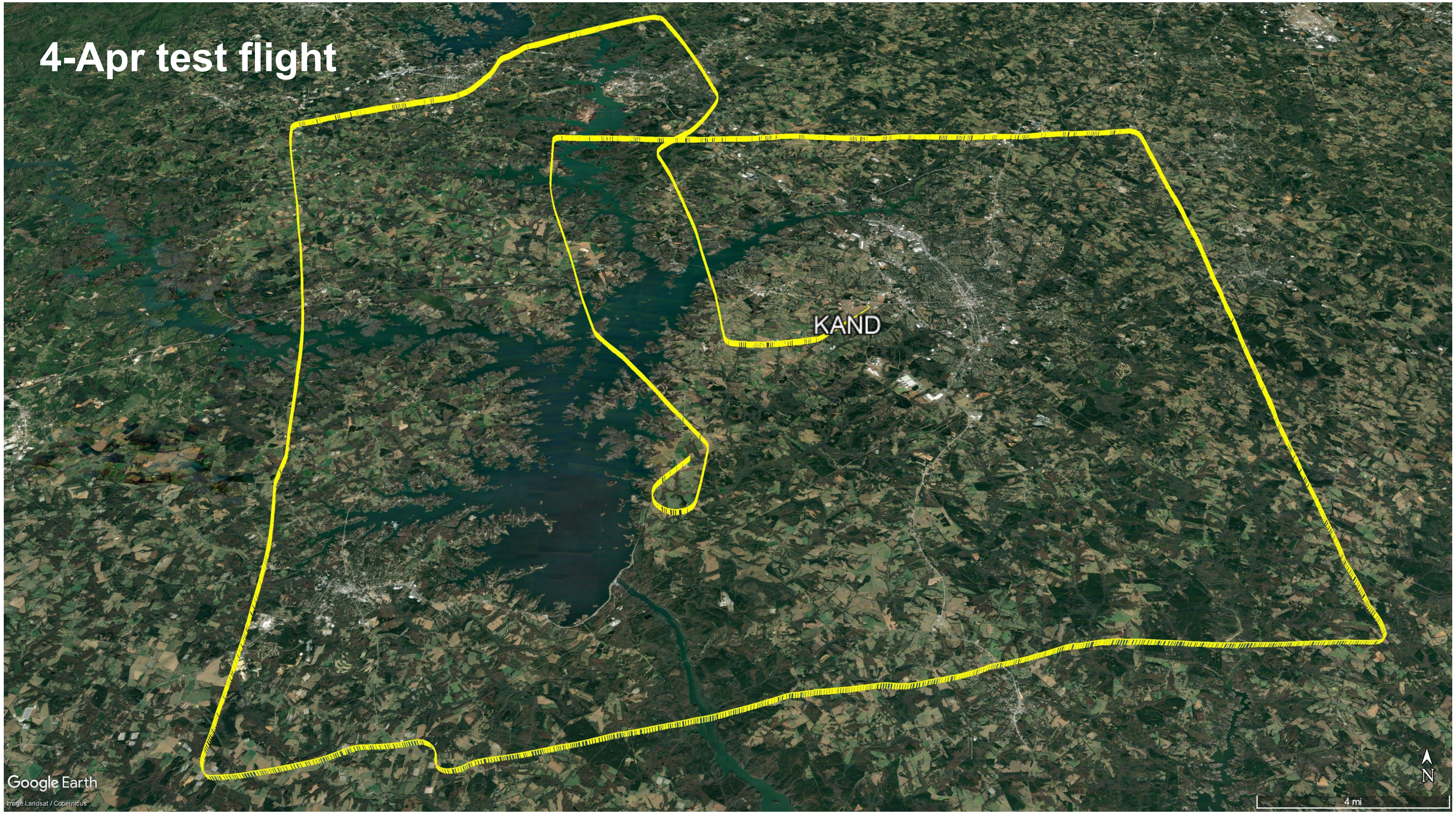
4-Apr test flight
KAND
Google Earth
Image Landsat / Copernicus
N
4 mi

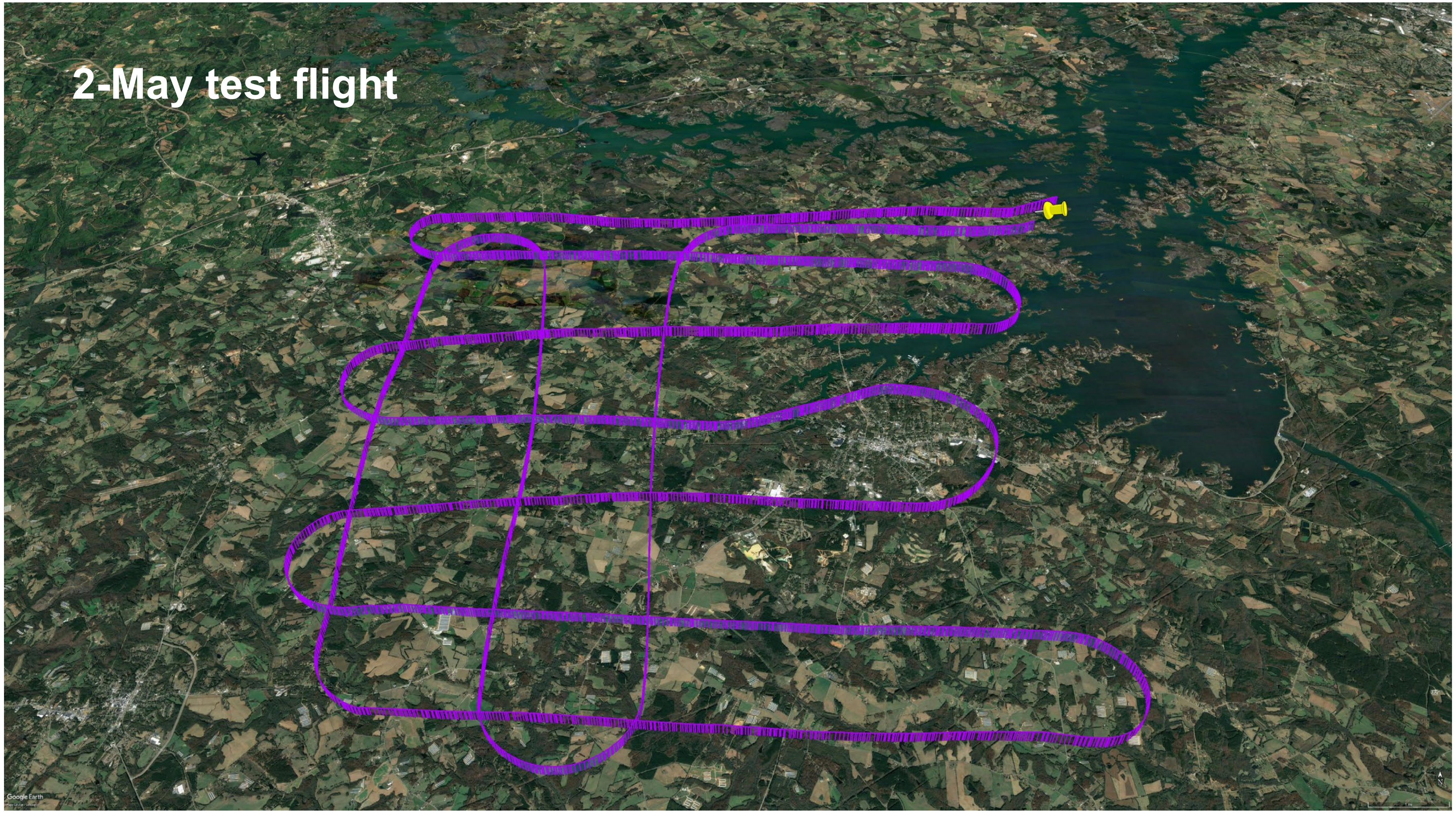
2-May test flight
Google Earth

Data S1.2

Research Survey Flights: Sampler performance data

Research Flight #	Date	Altitude ft MSL (m AGL)	Ave Flow, D1 (L·min^{-1})	Ave Flow, D2 (L·min^{-1})	Pressure, D1 (-kPa)	Pressure, D2 (-kPa)	Total Air Volume Filtered† L (m^3)
1	9-May	1800 (300)	10	10	42	42	1,500 (1.5)
2	9-May	4500 (1200)	10	13	51	55	1,725 (1.725)
3	9-May	8500 (2500)	11	7	55	48	1,350 (1.35)
4	4-Jun	1800 (300)	15	14	40	46	2,175 (2.175)
5	4-Jun	4500 (1200)	14	14	50	50	2,100 (2.1)
6	4-Jun	8500 (2500)	14	12	58	56	1,950 (1.95)

Data S1.2 Table 1. Sampler performance data for six dedicated research genetic survey flights conducted in the southeastern US. MSL: mean sea level, AGL: above ground level, Ave: average, D1/D2: duplicate 1/duplicate 2. †Total air volume was calculated as the product of airflow × sampling time.

Research Survey Flights: meteorological data

Research Flight #	Date	Sampling time, local (1h15min duration)	METAR_Zulu (UTC)	Wind Direction (°)	Wind Velocity (kts)	Air Temp (°C)	Dewpoint (°C)	Altimeter (in-Hg)	Density Altitude (ft)	Temp at Altitude (°C)
1	9-May	1837-1953	2225	080	05	23	08	3012	1700	14
2	9-May	1638-1754	2028	100	03	22	08	3013	1600	17
3	9-May	1429-1546	1811	VAR	03	20	07	3017	1300	11
4	4-Jun	1311-1426	1659	080	11G19	27	14	2992	2400	20
5	4-Jun	0944-1101	1328	040	10	23	14	2993	1900	15
6	4-Jun	0735-0850	1111	030	05	19	16	2992	1500	10

Research Flight #	Date	Altitude ft MSL (m AGL)	Ave GS (kts)	Distance (NM)	Remarks
1	9-May	1800 (300)	74	108	Wind shift SE in last 15 min of flight
2	9-May	4500 (1200)	85	104	Flew into dust plume from burn; Wind E
3	9-May	8500 (2500)	65	100	Wind N (at surface), E (at altitude)
4	4-Jun	1800 (300)	85	111	Light turbulence; thermals. Cloud bases 5,000 ft MSL. Wind ENE
5	4-Jun	4500 (1200)	76	106	Cloud bases 6,500ft MSL; WTA: 065°/07kts,11°C
6	4-Jun	8500 (2500)	81	105	WTA: 265°/07kts,6°C

Data S1.2 Table 2. Aeronautical and meteorological data for six dedicated research genetic survey flights conducted in the southeastern US: SM: statute miles, MSL: mean sea level, AGL: above ground level, Ave: average, GS: aircraft ground speed, NM: nautical miles, WTA: winds and temperatures aloft; VAR: variable (wind direction varies by 180° or more); G: gusting. Note: The wind direction given indicates that the wind comes in front of the aircraft when its heading equals the wind direction. When the wind is gusty with variations from the mean wind speed (gusts) exceeding 5 $m \cdot s^{-1}$ or 10 kts, speed variations are indicated after the mean wind direction and speed and preceded by the letter indicator "G" (for gusts).

Surface Analysis Prognostic Chart Forecasts (Continental US) for Research Surveys

9-May (3 flights)

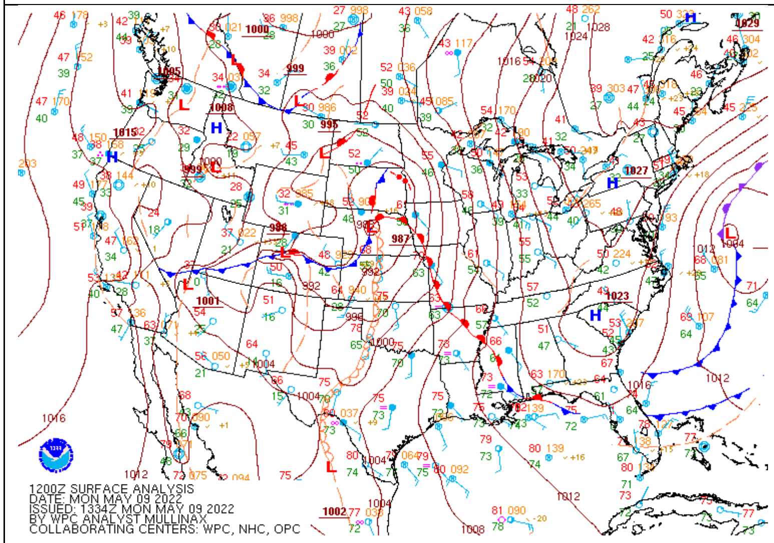

4-Jun (3 flights)

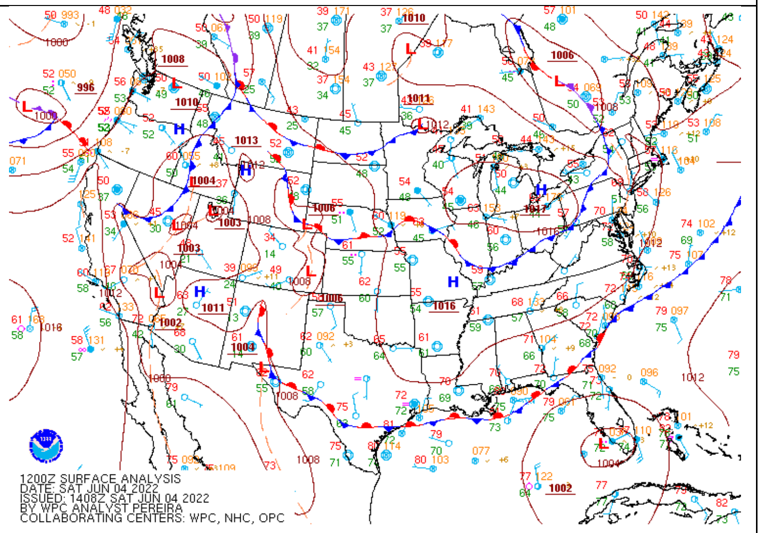

Data S1.2 Figure 1. Surface analysis prognostic chart forecasts for research survey flights: Surface analysis credit: © National Oceanic and Atmospheric Administration (NOAA).

Flight 1 (9-May)	AND,2022-05-09 22:25,M,M,M,70.00,5.00,M,30.12,M,10.00,M,CLR,KAND 092225Z AUTO 07005KT 10SM CLR 23/08 A3012 RMK T02300080<br>18A,2022-05-09 22:55,70.50,47.30,43.57,110.00,4.00,0.00,30.14,M,10.00,M,CLR,K18A 092255Z AUTO 11004KT 10SM CLR 21/09 A3014 RMK AO2 T02140085
Flight 2 (9-May)	AND,2022-05-09 20:25,M,M,M,90.00,5.00,M,30.13,M,10.00,M,CLR,KAND 092025Z AUTO 09005KT 10SM CLR 23/08 A3013 RMK T02300080<br>18A,2022-05-09 20:55,71.40,48.20,43.71,40.00,3.00,0.00,30.13,M,10.00,M,CLR,K18A 092055Z AUTO 04003KT 10SM CLR 22/09 A3013 RMK AO2 T02190090
Flight 3 (9-May)	AND,2022-05-09 18:10,M,M,M,350.00,2.00,M,30.17,M,10.00,M,CLR,KAND 091810Z AUTO 35002KT 10SM CLR 20/07 A3017 RMK T02000070 VRB03KT<br>18A,2022-05-09 18:55,68.90,46.80,45.16,120.00,7.00,0.00,30.17,M,10.00,M,CLR,K18A 091855Z AUTO 12007KT 080V140 10SM CLR 21/08 A3017 RMK AO2 T02050082
Flight 4 (4-Jun)	AND,2022-06-04 16:56,81.00,57.00,43.90,50.00,12.00,0.00,29.92,1012.30,10.00,19.00,FEW 5000,81.05,KAND 041656Z AUTO 05012G19KT 10SM FEW050 27/14 A2992 RMK AO2 SLP123 T02720139<br>18A,2022-06-04 17:35,78.60,58.60,50.30,90.00,10.00,0.00,29.92,M,10.00,16.00,BKN,4900.00,K18A 041735Z AUTO 09010G16KT 10SM BKN049 26/15 A2992 RMK AO2 T02590148
Flight 5 (4-Jun)	AND,2022-06-04 13:25,M,M,M,70.00,11.00,M,29.93,M,10.00,M,CLR,KAND 041325Z AUTO 07011KT 10SM CLR 23/14 A2993 RMK T02300140<br>18A,2022-06-04 14:15,73.60,60.10,62.68,60.00,5.00,0.00,29.94,M,10.00,M,SCT,6500.00,K18A 041415Z AUTO 06005KT 10SM SCT065 23/16 A2994 RMK AO2 T02310156
Flight 6 (4-Jun)	AND,2022-06-04 11:10,M,M,M,20.00,5.00,M,29.92,M,10.00,M,CLR,KAND 041110Z AUTO 02005KT 10SM CLR 19/16 A2992 RMK T01900160<br>18A,2022-06-04 11:35,65.30,63.50,93.90,50.00,5.00,0.00,29.92,M,10.00,M,CLR,K18A 041135Z AUTO 05005KT 10SM CLR 19/18 A2992 RMK AO2 T01850175

Data S1.2 Table 3. Meteorological aerodrome (METAR) data from automated surface [weather] observation systems (ASOS) for all research survey flights originating out of AND, Anderson Regional Airport (Anderson, SC) and during the flight at 18A, Franklin-Hart Airport (Canon, GA). *Data are provided in the following order: station, valid, tmpf, dwpf, relh, drct, sknt, p01i, alti, mslp, vsby, gust, skyc, metar; m=missing.* Airport meteorological automated surface weather observations (ASOS) METAR data codes can be referenced from NOAA at https://www.weather.gov/media/asos/aum-toc.pdf (accessed 4 August 2022).

Data S1.3

Supplemental Results: High-Throughput Amplicon Sequencing

Data S1.3 Figure 1. Relative abundance (%) of airborne bacterial phyla across the six research survey flights conducted in May at 300 m, 1200 m, and 2500 m, and in June at 300 m, 1200 m, and 2500 m. “Other” denotes that the highest taxonomic level was kingdom and resolution to phylum could not be achieved.

Data S1.3 Figure 1 is on the following page.

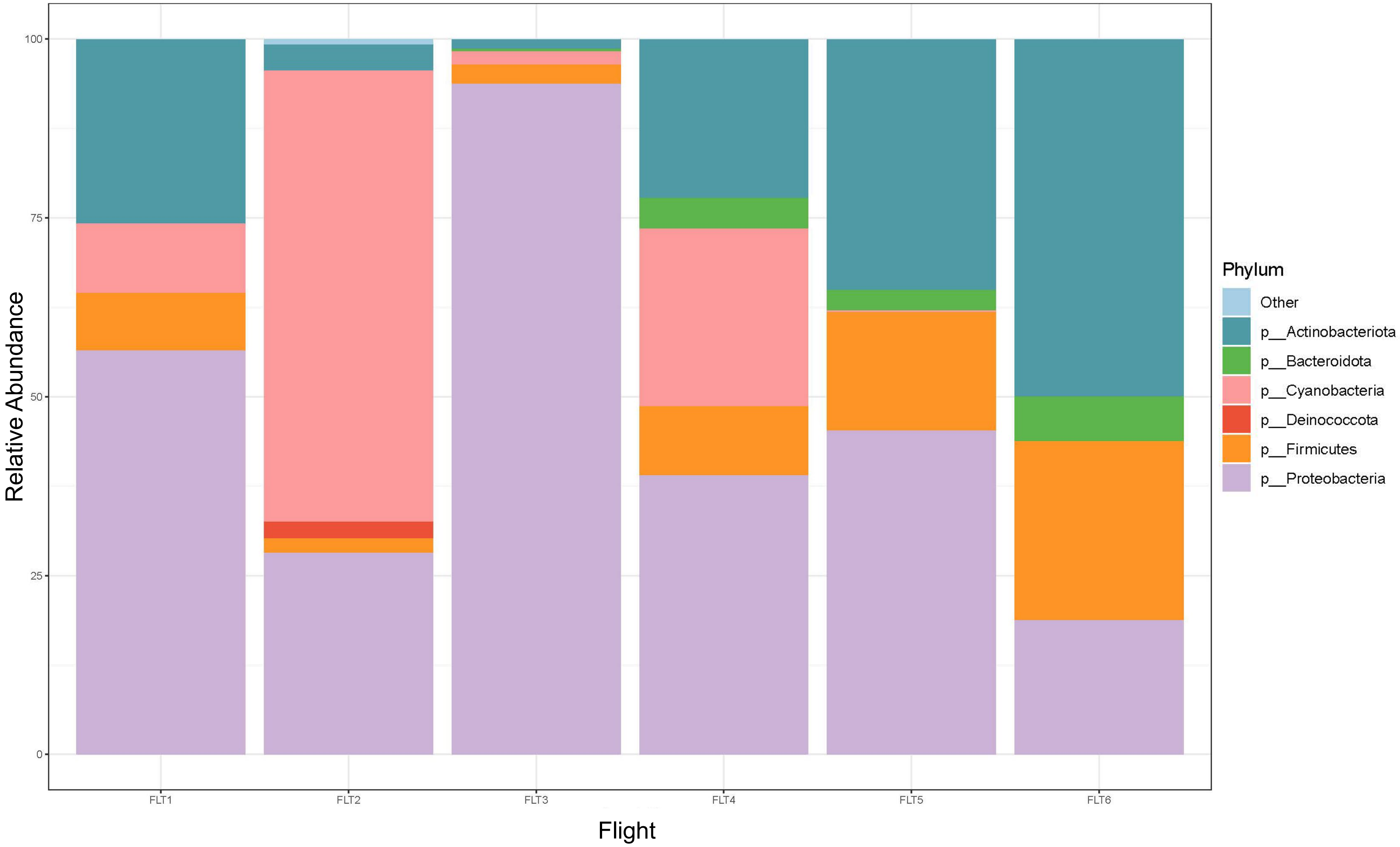

Relative Abundance
100
75
50
25
0
FLT1
FLT2
FLT3
FLT4
FLT5
FLT6
Flight
Phylum
Other
p__Actinobacteriota
p__Bacteroidota
p__Cyanobacteria
p__Deinococcota
p__Firmicutes
p__Proteobacteria

Data S1.3

Supplemental Results: High-Throughput Amplicon Sequencing

Data S1.3 Figure 2. Relative abundance (%) of airborne bacterial families detected on research flights combined in May (left panel) versus June (right panel). The question mark ("?") denotes airborne bacteria that could not be taxonomically identified to family level.

Data S1.3 Figure 2 is on the following page.

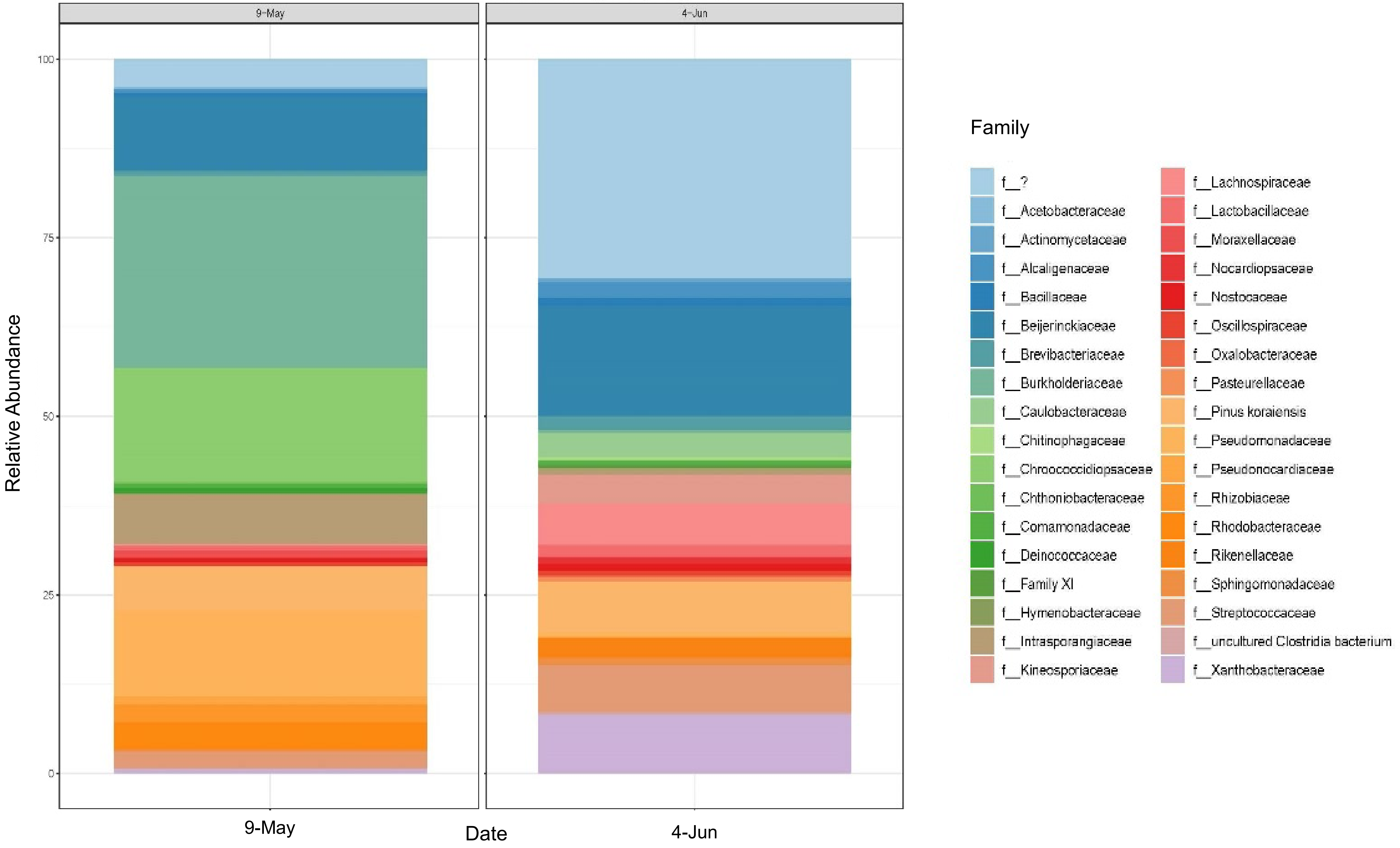
9-May
4-Jun
100
75
50
25
0
Relative Abundance
9-May
Date
4-Jun
Family
f__?
f__Acetobacteraceae
f__Actinomycetaceae
f__Alcaligenaceae
f__Bacillaceae
f__Beijerinckiaceae
f__Brevibacteriaceae
f__Burkholderiaceae
f__Caulobacteraceae
f__Chitinophagaceae
f__Chroococcidiopsaceae
f__Chthoniobacteraceae
f__Comamonadaceae
f__Deinococcaceae
f__Family XI
f__Hymenobacteraceae
f__Intrasporangiaceae
f__Kineosporiaceae
f__Lachnospiraceae
f__Lactobacillaceae
f__Moraxellaceae
f__Nocardiopsaceae
f__Nostocaceae
f__Oscillospiraceae
f__Oxalobacteraceae
f__Pasteurellaceae
f__Pinus koraiensis
f__Pseudomonadaceae
f__Pseudonocardiaceae
f__Rhizobiaceae
f__Rhodobacteraceae
f__Rikenellaceae
f__Sphingomonadaceae
f__Streptococcaceae
f__uncultured Clostridia bacterium
f__Xanthobacteraceae

Alpha Diversity within Bacterial (*16S*), Plant (*ITS*), and Vertebrate (*COI*) OTUs detected across six research survey flights:

Bacteria OTUs (*16S*)

SampleID	Shannon_Entropy	effective_Shannon_Entropy	Simpson_Index	effective_Simpson_Index	Richness	Altitude
FLT1	2.36	10.59	0.13	7.69	17	300m
FLT2	1.65	5.21	0.28	3.55	21	1200m
FLT3	1.38	3.97	0.39	2.55	33	2500m
FLT4	2.67	14.39	0.11	8.77	35	300m
FLT5	2.5	12.18	0.12	8.04	27	1200m
FLT6	2.13	8.44	0.14	7.11	10	2500m

Plant OTUs (*ITS*)

SampleID	Shannon_Entropy	effective_Shannon_Entropy	Simpson_Index	effective_Simpson_Index	Richness	Altitude
FLT1	1	2.73	0.48	2.08	4	300m
FLT2	0	1	1	1	1	1200m
FLT3	0.8	2.23	0.63	1.6	15	2500m
FLT4	0.16	1.17	0.95	1.06	5	300m
FLT5	0.05	1.05	0.99	1.01	3	1200m
FLT6	0.03	1.03	0.99	1.01	3	2500m

Vertebrate OTUs (*COI*)

SampleID	Shannon_Entropy	effective_Shannon_Entropy	Simpson_Index	effective_Simpson_Index	Richness	Altitude
FLT1	0.31	1.36	0.83	1.2	2	300m
FLT2	0.44	1.55	0.81	1.23	4	1200m
FLT3	0.86	2.35	0.5	2	3	2500m
FLT4	0.77	2.17	0.59	1.68	4	300m
FLT5	0.57	1.77	0.62	1.63	2	1200m
FLT6	0.53	1.7	0.66	1.52	3	2500m

Data S1.3 Table 1. Alpha Diversity within Bacterial (16S), Plant (ITS), and Vertebrate (COI) OTUs detected across six research survey flights. Five common alpha diversity measures are reported, including Shannon entropy and the Simpson index, together with their counterparts accounting for the effective number of species, as well as richness. SampleID represents the flight #.

Data S1.3

Supplemental Results: High-Throughput Amplicon Sequencing

Data S1.3 Figure 3. **Alpha Diversity Box Plots of Shannon Entropy (left panel) and Simpson indices (right panel) within Bacterial, Plant, and Vertebrate OTUs detected at altitudes of 300 m (blue), 1200 m (red), and 2500 m (purple).** Index value is on the y-axis; Altitude is on the x-axis.

Data S1.3 Figure 3 is on the following page.

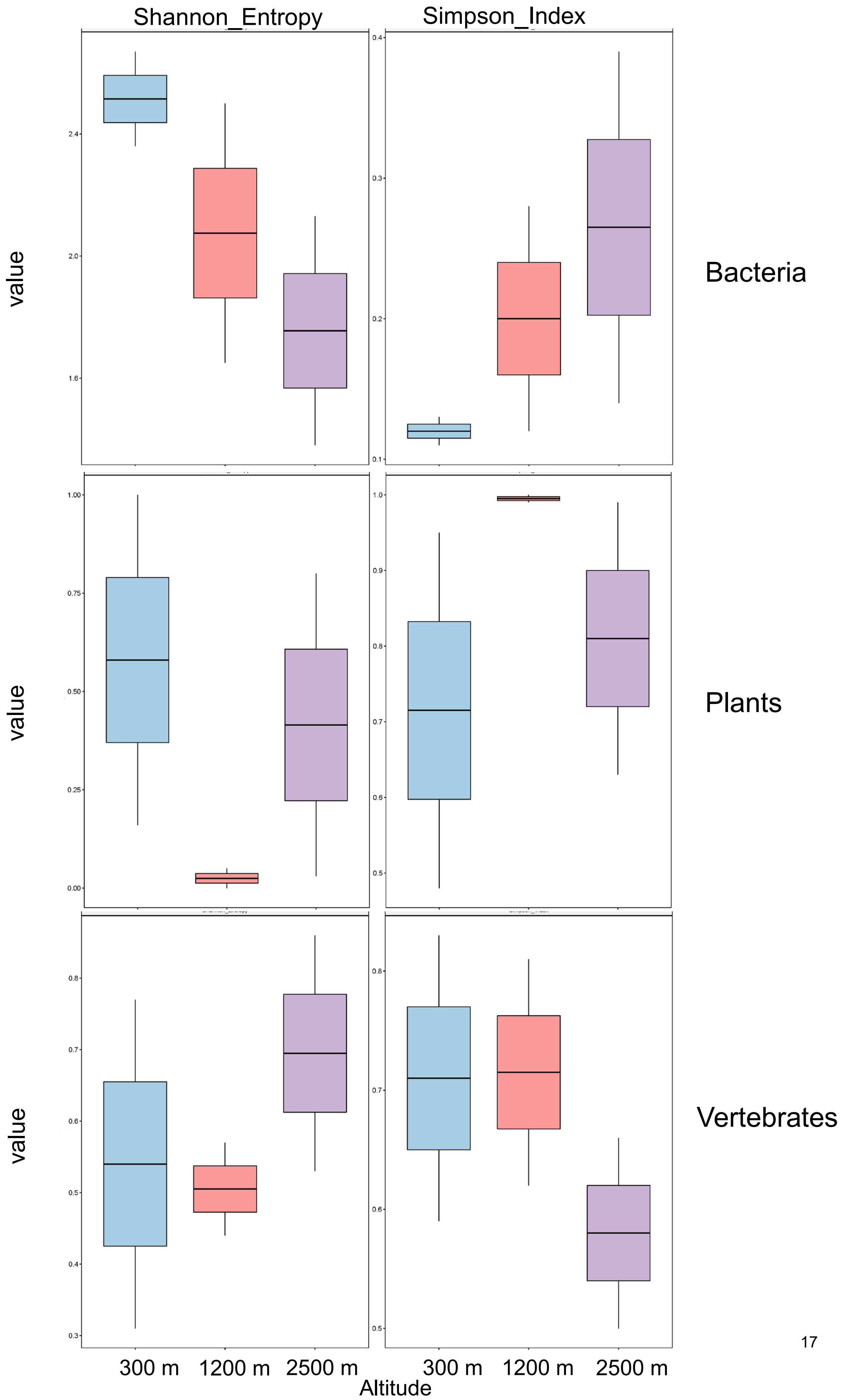
Shannon_Entropy
Simpson_Index
value
value
value
Bacteria
Plants
Vertebrates
300 m
1200 m
2500 m
300 m
1200 m
2500 m
Altitude

Data S1.3

Supplemental Results: High-Throughput Amplicon Sequencing

Data S1.3 Figure 4. Beta Diversity of Bacterial, Plant, and Vertebrate DNA Assemblages. Principal Coordinate Analysis (PCoA) plots for airborne bacteria, plant and vertebrate DNA by day and altitude of sampling. Ordination scatterplots are based on Bray-Curtis distance matrices. Colors indicate the day of sampling, while shapes indicate the altitude. The Permutational Multivariate Analysis of Variance (PERMANOVA) *p*-value for all groups indicates that samples from all groups are drawn from the same distribution (no groups are significantly different). Points that are grouped closer together represent OTU communities that are more similar in sequence composition. FLT=flight.

Data S1.3 Figure 4 is on the following page.

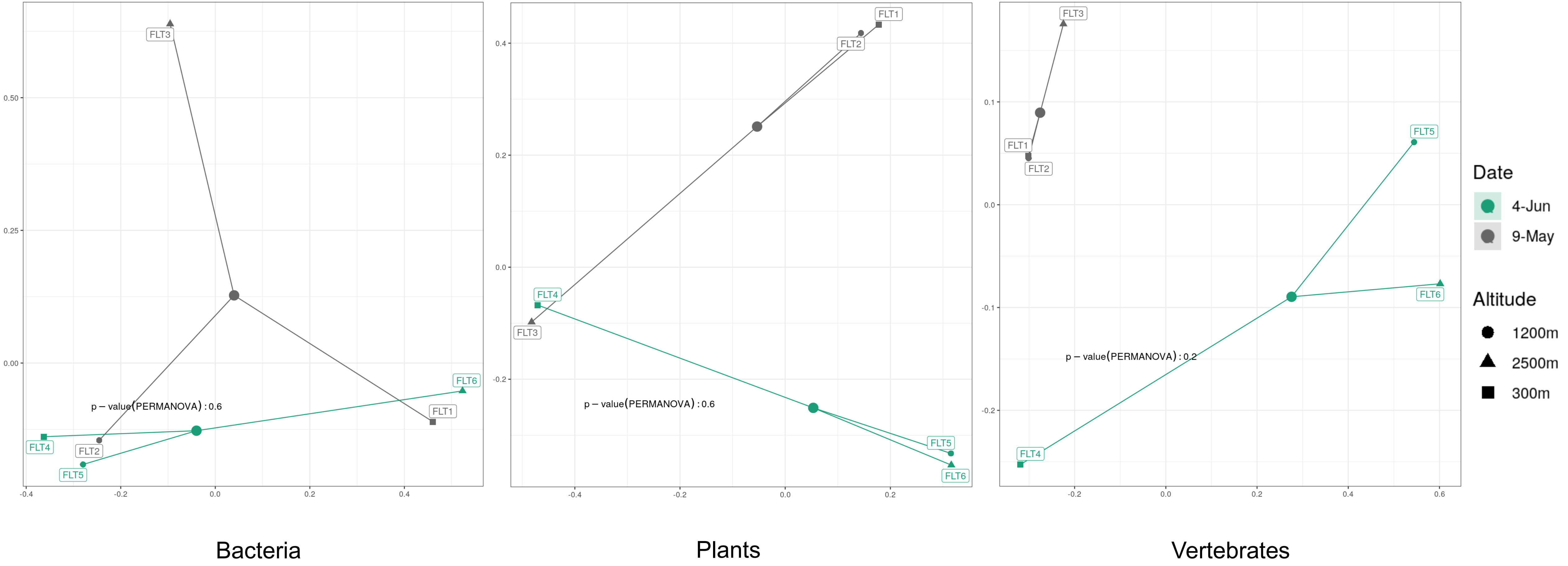
FLT3
FLT2
FLT1
FLT4
FLT5
FLT6
p – value(PERMANOVA) : 0.6
Bacteria
FLT1
FLT2
FLT4
FLT3
FLT5
FLT6
p – value(PERMANOVA) : 0.6
Plants
FLT3
FLT1
FLT2
FLT5
FLT6
FLT4
p – value(PERMANOVA) : 0.2
Vertebrates
Date
4-Jun
9-May
Altitude
1200m
2500m
300m

Data S1.4

PCR and Sanger sequencing verification of plant (*ITS +trnL*) and vertebrate (*COI+16S*) detections

To confirm vertebrate and plant representation by marker, Sanger sequencing was conducted on each DNA extract from lower biomass taxa (plants and vertebrates) using ABI Big Dye chemistry (Azenta Life Sciences, South Plainfield NJ) prior to high-throughput amplicon sequencing. Two sets of vertebrate/mammal-specific primers (*Kress and Erickson, 2012*) and two sets of plant-specific primers (*Taberlet et al., 2007*; *Johnson, Cox & Barnes, 2019a)* were used for Sanger sequencing.

Vertebrate *COI* PCR-Sanger: We amplified a portion of the cytochrome oxidase (*COI*) gene (5′ end) using Sanger sequencing M13-tagged AquaF2 and VR1d degenerate primers (*Ivanova, Clare & Borisenko, 2004*). This was the same primer used in the HTAS but with the Illumina® adapter exchanged for the M13-tagged Sanger adapter. PCR reagents consisted of 12.5 µL GoTaq® master mix (ProMega Corporation), 7.5 µL PCR-grade H_2O, 3 µL of template DNA, and 1 µL of each tagged primer (10 mM stocks of each) in 25 µL final volume. Reaction conditions were as follows: 95°C for 15 min, followed by 40 cycles of 94°C for 30 s, 51°C for 90 s, and 72°C for 90 s and final extension at 72°C for 10 min with a 10°C indefinite hold. PCRs were run in duplicate with positive (*Ovis aries*) and negative flight, extraction, and no template controls and gel purified prior to sequencing.

Mammal *16S* PCR-Sanger: We amplified a small portion of the mammal mitochondrial 16S gene using the primers mam1 and mam2 (*Taylor, 1996*). The PCR reagents consisted of 12.5 µL GoTaq® master mix (ProMega Corporation), 7.5 µL PCR-grade H_2O, 3 µL of template DNA, and 1 µL of each primer (10 mM stocks of each) in 25 µL final volume. Reaction conditions were as follows: 95°C for 15 min, 40 cycles of 94°C for 30 s, 55°C for 90 s, 72°C for 90 s, and final extension at 72°C for 10 min with a 10°C indefinite hold. PCRs were run in duplicate with positive (*O. aries)* and negative flight, extraction, and no template controls and gel purified prior to sequencing.

Plant *ITS* PCR-Sanger: We amplified a portion of the plant second internal transcribed spacer (*ITS2*) region in nuclear ribosomal RNA using the primers ITS2 and ITS4 (*White et al., 1990; Chen et al., 2010; Johnson, Cox & Barnes, 2019a*). This was the same *ITS* primer used in the HTAS without the partial Illumina® adapter. PCR reagents consisted of 12.5 µL GoTaq® master mix (ProMega Corporation), 7.5 µL PCR-grade H_2O, 3 µL of template DNA, and 1 µL of each primer (10 mM stocks of each) in 25 µL final volume. Reaction conditions were as follows: 95°C for 10 min, followed by 40 cycles of 95°C for 40 s, 49°C for 40 s, and 72°C for 40 s and final extension at 72°C for 5 min with a 4°C indefinite hold. PCRs were run in duplicate with

positive (*Arabidopsis thaliana*) and negative flight, extraction, and no template controls and gel purified prior to sequencing.

Plant *trnL* PCR-Sanger: We amplified an approximately 200 bp portion of the global chloroplast intron using Sanger M13-tagged primers TrnLF and TrnLR (*Taberlet et al., 2007*). The PCR reagents consisted of 12.5 µL GoTaq® master mix (ProMega Corporation), 7.5 µL PCR-grade H_2O, 3 µL of template DNA, and 1 µL of each primer (10 mM stocks of each) in 25 µL final volume. Reaction conditions for the PCR were as follows: 95°C for 10 min, followed by 32 cycles of 94°C for 2 min, 55°C for 1 min, and 72°C for 30 s and final extension at 72°C for 2 min with a 4°C indefinite hold. PCRs were run in duplicate with positive (*A. thaliana*) and negative flight, extraction, and no template controls and gel purified prior to sequencing.

Amplicons were sequenced in the 5′ direction for identification. Sequencing was repeated for samples where no priming, poor quality, homopolymeric or repetitive regions, or high background were encountered. Sanger sequences were queried in NCBI GenBank and aligned and trimmed in MEGA11: Molecular Evolutionary Genetics Analysis software (*Tamura et al., 2021*) using the MUSCLE (MUltiple Sequence Comparison by Log-Expectation) algorithm for varied sequence lengths. Multiple sequence alignments were visualized in SnapGene® version 6.1 (GSL Biotech, San Diego CA). Partial sequences for 16S large subunit ribosomal RNA (*16S*) have been deposited in NCBI under GenBank accession numbers OP314926-OP314929, and partial sequences for mitochondrial cytochrome c oxidase subunit I (*COI*) have been deposited in NCBI under GenBank accession numbers OP288123-OP288134). Partial sequences for internal transcribed spacer region 2 (*ITS2*) have been deposited in NCBI under GenBank accession numbers OP320462-OP320469, and partial sequences for the chloroplast tRNA-Leu (*trnL*) gene have been deposited in NCBI under BankIt accession numbers OP292304-OP292308.

Taxon	Gene Region	Primer	Primer Sequence
Vertebrate	*COI*	Forward: TSP_AquaF2	5′-GTAAAACGACGGCCAGATCACRACCATCATYAAYATRAARCC-3′
		Reverse: TSP_Vr1d	5′-CAGGAAACAGCTATGACTAGACTTCTGGGTGGCCRAARAAYCA-3′
Mammal	16S	Forward: 16Smam1	5′-CGGTTGGGGTGACCTCGGA-3′
		Reverse: 16Smam2	5′-GCTGTTATCCCTAGGGTAACT-3′
Plant	P6 loop intron	Forward: TSP_trnLF	5′-GTAAAACGACGGCCAGCGAAATCGGTAGACGCTACG-3′
		Reverse: TSP_trnLR	5′-CAGGAAACAGCTATGACCCATTGAGTCTCTGCACCTATC-3′
Plant	Global nucleic	Forward: ITS2	5′-ATGCGATACTTGGTGTGAAT-3′
		Reverse: ITS4	5′-TCCTCCGCTTATTGATATGC-3′

Data S1.4 Table 1. Primers used for amplification confirmation prior to HTAS. COI: cytochrome *c* oxidase subunit I; 16S: mitochondrial 16 ribosomal RNA; TSP: tagged sequencing primer; ITS2: internal transcribed spacer region 2. Underline denotes untagged primer.

Kress WJ, Erickson DL (eds.). 2012.DNA Barcoding in Mammals. In: Methods in Molecular Biology. Totowa, NJ: Humana Press. DOI: 10.1007/978-1-61779-591-6.

Taberlet P, Coissac E, Pompanon F, Gielly L, Miquel C, Valentini A, Vermat T, Corthier G, Brochmann C, Willerslev E. 2007. Power and limitations of the chloroplast trnL (UAA) intron for plant DNA barcoding. *Nucleic Acids Research* 35:e14–e14. DOI: 10.1093/nar/gkl938.

Tamura K, Stecher G, Kumar S. 2021. MEGA11: Molecular Evolutionary Genetics Analysis Version 11. *Molecular Biology and Evolution* 38:3022–3027. DOI: 10.1093/molbev/msab120.

Taylor PG. 1996. Reproducibility of ancient DNA sequences from extinct Pleistocene fauna. *Molecular Biology and Evolution* 13:283–285. DOI: 10.1093/oxfordjournals.molbev.a025566.

COI **(OP288123-OP288134):**

Submission	SeqID	Accession number	Organism	Altitude (meters AGL)
SUB11964631	Seq12	OP288123	*G. gallus*	300m
SUB11964631	Seq13	OP288124	*G. gallus*	300m
SUB11964631	Seq14	OP288125	*G. gallus*	2500m
SUB11964631	Seq15	OP288126	*G. gallus*	2500m
SUB11964631	Seq16	OP288127	*G. gallus*	2500m
SUB11964631	Seq17	OP288128	*G. gallus*	2500m
SUB11964631	Seq18	OP288129	*B. taurus*	1200m
SUB11964631	Seq19	OP288130	*B. taurus*	1200m
SUB11964631	Seq20	OP288131	*B. taurus*	2500m
SUB11964631	Seq21	OP288132	*B. taurus*	2500m
SUB11964631	Seq22	OP288133	*B. taurus*	2500m
SUB11964631	Seq23	OP288134	*B. taurus*	2500m

16S **(OP314926-OP314929):**

SUB11965351	Seq1	OP314926	*B. taurus*	300m
SUB11965351	Seq2	OP314927	*B. taurus*	300m
SUB11965351	Seq3	OP314928	*B. taurus*	2500m
SUB11965351	Seq4	OP314929	*B. taurus*	2500m

ITS **(OP320462-OP320469):**

SUB11964727	Seq24	OP320462	*Quercus*	1200m
SUB11964727	Seq25	OP320463	Fagales	2500m
SUB11964727	Seq26	OP320464	Fagales	2500m
SUB11964727	Seq27	OP320465	*Allium sativum*	300m
SUB11964727	Seq28	OP320466	Didymellaceae; *Neoascochyta*	300m
SUB11964727	Seq29	OP320467	Didymellaceae; *Neoascochyta*	1200m
SUB11964727	Seq30	OP320468	Didymellaceae; *Neoascochyta*	2500m
SUB11964727	Seq31	OP320469	Didymellaceae; *Neoascochyta*	2500m

trnL **(OP292304-OP292308):**

BankIt2615691	Seq32	OP292304	*Quercus*	2m (ground test)
BankIt2615691	Seq33	OP292305	*Quercus*	300m
BankIt2615691	Seq34	OP292306	*Quercus*	300m
BankIt2615691	Seq35	OP292307	*Pinus*	1200m
BankIt2615691	Seq36	OP292308	*Picea/Pinus*	1200m

Data S1.4 Table 2. GenBank Sanger sequence data from this study. Confirmatory Sanger DNA sequences are deposited in NCBI under the following GenBank accession numbers: OP288123-OP288134 (*COI*), OP314926-OP314929 (*16S*), OP320462-OP320469 (*ITS*), and OP292304-OP292308 (*trnL*), including organism and altitude from which airborne DNA was collected.
